# Generating Brain Waves, the Power of Astrocytes

**DOI:** 10.3389/fnins.2019.01125

**Published:** 2019-10-18

**Authors:** Yossi Buskila, Alba Bellot-Saez, John W. Morley

**Affiliations:** ^1^School of Medicine, Western Sydney University, Campbelltown, NSW, Australia; ^2^International Centre for Neuromorphic Systems, The MARCS Institute, Western Sydney University, Penrith, NSW, Australia

**Keywords:** brain waves, oscillations, astrocytes, spatial buffering, K^+^ clearance

## Abstract

Synchronization of neuronal activity in the brain underlies the emergence of neuronal oscillations termed “brain waves”, which serve various physiological functions and correlate with different behavioral states. It has been postulated that at least ten distinct mechanisms are involved in the formulation of these brain waves, including variations in the concentration of extracellular neurotransmitters and ions, as well as changes in cellular excitability. In this mini review we highlight the contribution of astrocytes, a subtype of glia, in the formation and modulation of brain waves mainly due to their close association with synapses that allows their bidirectional interaction with neurons, and their syncytium-like activity via gap junctions that facilitate communication to distal brain regions through Ca^2+^ waves. These capabilities allow astrocytes to regulate neuronal excitability via glutamate uptake, gliotransmission and tight control of the extracellular K^+^ levels via a process termed K^+^ clearance. Spatio-temporal synchrony of activity across neuronal and astrocytic networks, both locally and distributed across cortical regions, underpins brain states and thereby behavioral states, and it is becoming apparent that astrocytes play an important role in the development and maintenance of neural activity underlying these complex behavioral states.

## Introduction

### Neuronal Oscillations

In the central nervous system (CNS), neurons communicate via electrochemical signals which leads to flow of ionic currents through synaptic contacts (Schaul, 1998). At the network level, the synchronization of the neuron’s electrical activity gives rise to rhythmic voltage fluctuations traveling across brain regions, known as neuronal oscillations or brain waves (Buzsaki, 2006).

Neuronal oscillations can be modulated in space and time and are affected by the dynamic interplay between neuronal connectivity patterns, cellular membrane properties, intrinsic circuitry, speed of axonal conduction and synaptic delays (Nunez, 1995; Sanchez-Vives and McCormick, 2000; Cunningham et al., 2006; Buskila et al., 2013; Tapson et al., 2013). At the cellular level, these synchronous oscillations fluctuate between two main states, known as “up states” and “down states”, which occur in the neocortex both *in vitro* and *in vivo* (Sanchez-Vives and McCormick, 2000). Whereas Down states refer to resting activity and membrane hyperpolarization, Up states are associated with neuronal depolarization and firing bursts of action potentials (Cossart et al., 2003). Importantly, Up states occurring within spatially organized cortical ensembles have been postulated to interact with each other to produce a temporal window for neuronal network communication and coordination (Fries, 2005). This network coherence was found to be essential for several sensory and motor processes, as well as for cognitive flexibility (i.e., attention, memory), thereby playing a fundamental role in the brain’s basic functions (Fries et al., 2001; Tallon-Baudry et al., 2004).

Emerging technologies during the past decades led to the description of multiple neuronal oscillations displaying different electrophysiological and connectivity properties across brain areas including the neocortex, thalamus and hippocampus (Steriade, 2006). Using power spectrum analysis, investigators identified that neuronal oscillations fluctuate within specific frequency bands, ranging from very slow (<0.01 Hz) to ultra-fast (>1,000 Hz) oscillations, mediated by at least ten different mechanisms (Penttonen and Buzsáki, 2003). Whereas fast oscillators are found to be more localized within a restricted neural volume (Contreras and Llinas, 2001), slow oscillations typically involve large synchronous membrane voltage fluctuations in wider areas of the brain (He et al., 2008). These network dynamics and connectivity patterns can change according to the behavioral state, with some frequency bands being associated with sleep, while other frequencies predominate during arousal or conscious states (Brooks, 1968; Achermann and Borbély, 1997; Murthy and Fetz, 2006) (Table 1). Interestingly, neuronal oscillations interact across different frequency bands to modulate each other and engage specific behaviors (Buzsaki, 2006; Steriade, 2006), and previous studies have postulated that different oscillation frequencies either compete with each other or cooperate in a specific manner to participate in distinct physiological processes such as bias of input selection, temporal linkage of neurons into assemblies and facilitation of synaptic plasticity (Buzsáki and Draguhn, 2004; Isomura et al., 2006). Moreover, oscillation phase relationships between regions are diverse and can be modulated by sensory and motor experiences (Maris et al., 2016), thereby adding greater complexity in deciphering how brain waves coordinate to subserve important functions in both the developing and adult human brain.

**TABLE 1:** Common characteristics of brain waves.

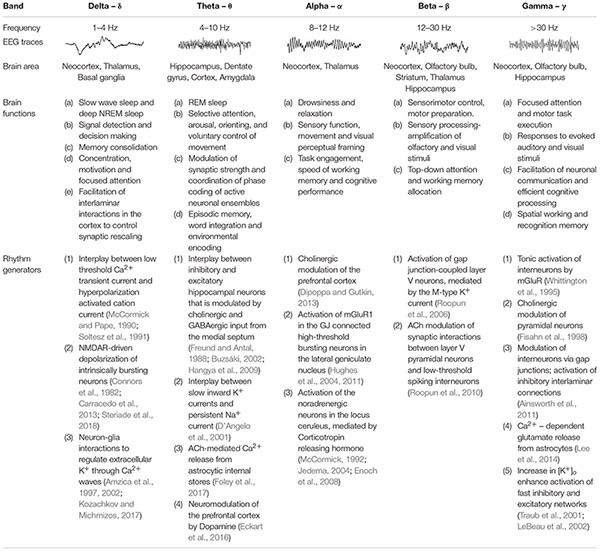

The common view of oscillatory frequency bands is that they represent groups of neuronal oscillations acting as distinct entities that work similarly during particular brain functions (Watson, 2015), and therefore, can serve as a fundamental tool for both clinical diagnosis and brain research (Huber et al., 2004; Buzsaki, 2006). In addition, the fact that brain waves expressed in many species (e.g., human, macaque, cat, rabbit, rat) and their behavioral correlates are preserved throughout evolution is a testament to their fundamental role in mediating synchronization across neuronal ensembles to efficiently coordinate and propagate neuronal signals at the network level (Hughes et al., 2004; Bereshpolova et al., 2007; Skaggs et al., 2007; Nir et al., 2011; Peyrache et al., 2011).

### Mechanisms Underpinning Neuronal Oscillations

Neuronal oscillations show a linear progression on a natural logarithmic scale with little overlap (Penttonen and Buzsáki, 2003), leading to the suggestion that at least ten distinct and independent mechanisms are required to cover the large frequency range of brain waves, and it has been reported that several oscillations are driven by multiple mechanisms (Buzsáki and Draguhn, 2004; Buzsaki, 2006). Some of the suggested mechanisms underlying the generation of network oscillations are summarized in Table 1, and most of them include reciprocal interactions between excitatory and inhibitory mechanisms (Singer, 1993) or changes in cellular excitability (Liljenström and Hasselmo, 1993; Ainsworth et al., 2011; Bellot-Saez et al., 2018). The latter is often associated with alterations in extracellular ions (e.g., K^+^; Ca^2+^) and the hyperpolarization-activated inward current (*I*_*h*_) (Steriade et al., 1993), which can regulate intrinsic membrane properties such as the resonance frequency (Tohidi and Nadim, 2009; Bellot-Saez et al., 2018), as well as the strength and frequency of network oscillations (Yue and Huguenard, 2001). In this mini-review we will focus on mechanisms by which astrocytes effect neuronal excitability.

Neurons consist of inherent membrane resonance and frequency preference properties (Hutcheon and Yarom, 2000; Buskila et al., 2013) that allow them to act as resonators or transient oscillators that amplify inputs within certain frequencies (Alonso and Llinás, 1989). This oscillatory behavior at multiple frequencies depends on the accurate combination of both low-pass (i.e., passive leak conductance, membrane capacitance) and high-pass (i.e., voltage-gated channels activated close to the resting membrane potential, RMP) filtering properties (Buzsaki, 2006), which endow neurons with a wide repertoire to respond faster and more efficiently to spike trains or fast inputs (Pike et al., 2000). Therefore, alterations in membrane conductance or excitability along the somatodendritic compartments result in differential tuning of the resonant response in different cell types (e.g., interneurons vs. pyramidal or cholinergic cells), which on the one hand filter inputs from neurons that are not synchronized [see Hutcheon and Yarom (2000) and Laudanski et al. (2014) for comprehensive review], and on the other hand is essential for the synchronization of neurons that express similar resonance, therefore, sculpting the functionality of a neuronal network (Hutcheon and Yarom, 2000; Whittington and Traub, 2003; Laudanski et al., 2014; Kékesi et al., 2019).

Consequently, changes in the concentration of extracellular ions that impact the excitability and resonance behavior of individual neurons (e.g., K^+^, Mg^2+^, Ca^2+^), can affect brain rhythms. Indeed, a recent comprehensive report from Nedergaard’s group, in which they have recorded different brain rhythms during the sleep-awake cycle show that different rhythms are linked with alterations in extracellular concentrations of K^+^, Ca^2+^, Mg^2+^, and H^+^ (Ding et al., 2016), confirming that cellular mechanisms which particularly affect the ionic composition of the extracellular fluid can modulate the excitability and synchronous activity of neurons, thus affecting the different brain rhythms. Accordingly, K^+^ channels which mediate K^+^ efflux and membrane repolarization, play a crucial role in determining the overall network excitability and have been suggested to affect the generation of neuronal oscillations at multiple frequencies (Buzsaki, 2006). Consistent with this view, D’Angelo et al. (2001) showed via experimental and computational modeling of cerebellar granule cells that slow repolarizing K^+^ currents terminate the oscillatory “up state” of theta oscillations amplified by a persistent Na^+^ current and therefore, underlie the bursting and resonant behavior of theta oscillations. In line with these results, activation of K^+^ currents has been associated with enhanced spike timing precision at gamma frequencies in both pyramidal and basket cells in the hippocampus (Penttonen et al., 1998), as well as with lower frequency oscillations in the delta range (Ushimaru et al., 2012). Moreover, intracellular recordings of cortical neurons during alterations in K^+^ homeostasis indicate changes in neuronal excitability and resonance behavior that affected the amplification of network oscillations (Bellot-Saez et al., 2018).

**FIGURE 1:**
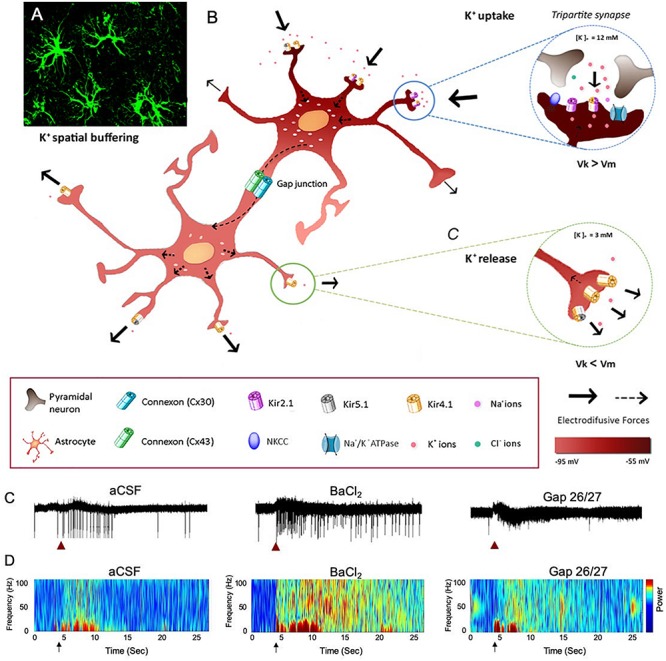
The impact of astrocytic K^+^ clearance on network oscillations. **(A)** Image of GFP labeled cortical astrocytes depicting their organization in non-overlapping domains. **(B)** Schematic diagram describing the mechanisms of astrocytic K^+^ clearance. Top-right inset – *K*^+^ *uptake-* local increase of [K^+^]_*o*_ is cleared from the extracellular space through the astrocytic Kir channels, NKCC and Na^+^/K^+^ ATPase. Eventually, K^+^ ions flow intracellularly through GJ-connected astrocytes (K^+^ spatial buffering) and promote a distal outward current to the extracellular space, where [K^+^]_*o*_ is low (∼3 mM) as shown in the lower inset (K^+^ release). Arrows indicate the direction of K^+^ driving force. **(C)** The functional role of astrocytic K^+^ clearance processes on network oscillations. Traces of extracellular recordings showing the network activity before and after brief (1 s) application of 30 mM KCl (red arrow), in normal aCSF (left) and after bath application of 100 μM BaCl_2_ (selective blocker of astrocytic Kir4.1 channels, middle trace) or Gap-26/27 (selective blocker of Cx43, right). Note the increase in network excitability following the increase in [K^+^]_*o*_ depicted as increase in spiking activity. **(D)** Color coded spectrogram of network oscillations depicting the network activity before and after local increase in [K^+^]_*o*_ (black arrows, imitating high local neuronal activity) under normal conditions (aCSF, left), following impairment in K^+^ uptake with 100 μM BaCl_2_ (middle spectrogram) or following blockade of astrocytic spatial buffering with selective astrocytic gap-junction blockers (GAP-26/27, right). Adapted from Neuroscience and Biobehavioral Reviews, vol 77, Alba Bellot-Saez, Orsolya Kékesi, John W. Morley, and Yossi Buskila, Astrocytic modulation of neuronal excitability through K^+^ spatial buffering, 87–97, copyright (2017), with permission from Elsevier Ltd., under CC BY license (http://creativecommons.org/licenses/by/4.0/).

K^+^ homeostasis in the brain is governed by the activity of astrocytes through several mechanisms, including K^+^ clearance from the extracellular fluid. Astrocytes are strategically located close to synapses, which allows them to critically regulate the overall network function (Wang et al., 2012; Bellot-Saez et al., 2017). Two major mechanisms of astrocytic K^+^ clearance have been established: (i) net K^+^ uptake, in which the excess of extracellular K^+^ ([K^+^]_*o*_) is taken up by K^+^ cotransporters (Na^+^/K^+^/2Cl^–^), Na^+^/K^+^ pumps (Na^+^/K^+^ ATPase), and inward rectifying K^+^ channels (K^+^_*ir*_) that are expressed in astrocytic processes and (ii) K^+^ spatial buffering, in which K^+^ ions propagate from high to low concentrations through gap-junction (GJ) mediated astrocytic networks by employing membrane voltage differences between the local K^+^ reversal potential to the astrocytic network membrane potential, and then released in distal regions of the astrocytic networks (Figure 1). Ultimately, the [K^+^]_*o*_ is returned to baseline levels to prevent hyperexcitability (Verkhratsky and Nedergaard, 2018). Consistent with the importance of the K^+^ clearance to normal oscillatory functioning, genetically modified mice that suffer from impaired clearance mechanisms exhibit epileptic seizures, growth retardation, and premature lethality at the age of 2 weeks (Kofuji et al., 2000; Bellot-Saez et al., 2017; Do-Ha et al., 2018). However, recent reports indicate that under physiological conditions, neuromodulators can directly trigger an increase in [K^+^]_*o*_ and thus signal through astrocytes to alter neural circuit activity and regulate network oscillations (Ding et al., 2016; Ma et al., 2016).

### Astrocytic Modulation of Brain Waves

Numerous studies revealed the essential contributions made by astrocytes to many physiological brain functions, including synaptogenesis (Ullian et al., 2001), metabolic coupling (Magistretti, 2006), nitrosative regulation of synaptic release (Buskila et al., 2005; Abu-Ghanem et al., 2008; Buskila and Amitai, 2010), synaptic transmission (Fields and Stevens-Graham, 2002), network oscillations (Bellot-Saez et al., 2018), and plasticity (Suzuki et al., 2011; Oberheim et al., 2012).

Astrocytes express a plethora of receptors, ion channels, pumps (i.e., ATPase) and cotransporters allowing them to dynamically interact with neurons through several pathways (Haydon and Carmignoto, 2006; Giaume and Theis, 2010; Larsen and Macaulay, 2014). Despite lacking the ability to fire action potentials, astrocytes communicate with neurons and other astrocytes mainly via Ca^2+^ signals (Cornell-Bell et al., 1990; Shigetomi et al., 2010). Astrocytic Ca^2+^ signals can occur both independently of neuronal activity or following neurotransmitter release and include intrinsic Ca^2+^ oscillations within individual cells and Ca^2+^ waves that propagate from one astrocyte to another (Zur Nieden and Deitmer, 2006; Nett et al., 2017). Indeed, recent studies found that astrocytic Ca^2+^ signaling and glutamate clearance by astrocytes play an essential role in the regulation of the network activity and K^+^ homeostasis, which ultimately affects the neuronal excitability underlying network oscillations (Wang et al., 2012; Ding et al., 2016). Recently, Ma et al. (2016) showed that neuromodulators can signal through astrocytes by affecting their Ca^2+^ oscillations to alter neuronal circuitry and consequently behavioral output. In line with these observations, Nedergaard’s group further demonstrated that bath application of neuromodulators to cortical brain slices increased [K^+^]_*o*_ regardless of synaptic activity (Ding et al., 2016), suggesting that increased [K^+^]_*o*_ could serve as a mechanism to maximize the impact of neuromodulators on the synchronous activity of neurons and their recruitment into networks.

Interestingly, an *in vivo* study found that spontaneous Ca^2+^ oscillations in astrocytes differ between cortical layers, suggesting functional network segregation imposed by astrocytic function (Takata and Hirase, 2008). Indeed, the spatial and functional organization of astrocytes varies between different brain regions (Houades et al., 2008; Chai et al., 2017; Matias et al., 2019) establishing that astrocytes are organized into anatomical and functional compartments (Pannasch and Rouach, 2013). Similarly, a computational model of three-dimensional astrocytic networks showed that the propagation of astrocytic Ca^2+^ waves is highly variable between brain regions depending on their GJ-coupling organization within the astrocytic network, with short-distance connections favoring spreading of Ca^2+^ waves over wider areas (Lallouette et al., 2014). In addition, several studies have provided evidence that astrocytes respond to different neuronally released neurotransmitters and neuromodulators (e.g., Acetylcholine, 5-HT, Histamine, Norepinephrine, Dopamine) by eliciting Ca^2+^ elevations that trigger signaling cascades leading to alterations in the concentrations of intracellular and extracellular ions (e.g., Na^+^, Ca^2+^, K^+^) and gliotransmitter release (Blomstrand et al., 1999; Jung et al., 2000; Oikawa et al., 2005; Ding et al., 2013; Jennings et al., 2017; Covelo and Araque, 2018). These studies emphasize the bidirectional communication pathway between neurons and astrocytes, which establish a synergetic mechanism to affect network oscillations.

Recently, Mariotti et al. (2016, 2018) demonstrated that astrocytic modulation and signaling are circuit-specific, as cortical astrocytes not only respond to excitatory inputs, but also react to inhibitory interneurons by eliciting weak or strong [Ca^2+^]_*i*_ elevations. In addition, two-photon imaging experiments revealed that cortical astrocytes are fast enough to respond to sensory stimulation by evoking fast Ca^2+^ events (Stobart et al., 2018). Together, these studies suggest that astrocytes are able to process different patterns of network activity with a variety of Ca^2+^ signals in order to decode and integrate local synaptic activity and plasticity (Perea and Araque, 2007; Henneberger et al., 2010; Navarrete et al., 2012), as well as other physiological processes including vasodilation through nitric oxide (Buskila and Amitai, 2010; Muñoz et al., 2015), K^+^ signaling (Filosa et al., 2006), release of trophic factors (Igelhorst et al., 2015), and inflammatory mediators (Michelucci et al., 2016). Moreover, gliotransmitters can activate neuronal receptors, including extrasynaptic NR1/NR2B-containing NMDA receptors (Fellin et al., 2004; Jourdain et al., 2007; Wang et al., 2013), thereby establishing reciprocal interactions between neurons and astrocytes that result in the overall modulation of the network excitability and synchronous activity of groups of neurons (Sardinha et al., 2017; Adamsky et al., 2018).

Astrocytes mediate long distance communication not only via Ca^2+^ waves but also through ATP release (Haas et al., 2006; Suadicani, 2006), which is followed by its degradation to adenosine by extracellular nucleotidases, leading to synaptic inhibition of neurotransmission (Pascual et al., 2005). Consistently, ATP release from neocortical astrocytes has been found to activate purinergic currents in pyramidal neurons, followed by attenuation of synaptic and tonic inhibition (Lalo et al., 2014). These results suggest that cortical astrocytes, via exocytosis of ATP, could also play a role in the modulation of neuronal GABA release and thus phasic and tonic inhibition, which eventually contribute to the generation of hypersynchronous oscillations at the network level.

## Discussion

In the 19th century, Carl Ludwig Schleich was first to propose that neuroglia is the anatomical locus for controlling neuronal excitation and its transmission from neuron to neuron (Schleich, 1894; Dierig, 1994). A year later, Ramón y Cajal, the father of modern neuroscience, proposed that astrocytes are directly involved in modulating neuronal activity by isolating neighboring neurons (Cajal, 1895; Navarrete and Araque, 2014). In support of this view, Cajal further revealed that “the neuroglia is abundant where intercellular connections are numerous and complicated, not due to the existence of contacts, but rather to regulate and control them, in such a manner that each protoplasmic expansion is in an intimate relationship with only a particular group of nerve terminal branches”, which led him to propose that astrocytes exert a major role in modulating brain function during different behavioral states (Cajal, 1895, 1897). More than a century later, with the development of powerful electrophysiological and imaging tools (Berger et al., 2007; Pál et al., 2015), these initial insights about astrocytes as potential modulators of the brain circuitry are gaining more support.

The close association of astrocytes with synapses led to the concept of the tripartite synapse, (consisting the pre-synaptic terminal, the post-synaptic membrane and the cradling astrocyte) which allows the bidirectional interaction of astrocytes with neurons (Araque et al., 1999). Although the molecular and cellular pathways in which astrocytes affect neuronal network activity and brain rhythms are not fully clear, numerous *in vivo* and *in vitro* studies indicate that they are playing a key role in the modulation of neuronal excitability and network synchronous activity, thereby contribute to the “conversation in the brain” (Verkhratsky and Nedergaard, 2018).

The fact that astrocytes can regulate the activity of individual neurons prompted a new concept of network modulation termed “lateral astrocyte synaptic regulation” (Covelo and Araque, 2016). Accordingly, astrocytic regulation of synaptic transmission is heterosynaptic and not restricted to the active synapse itself, but involving the activity of distant tripartite synapses via paracrine signaling of gliotransmitters that depends on the morphological and functional properties of astrocytes, thereby acting as a syncytium that can influence neuronal properties over wide brain regions (Pirttimaki et al., 2017). However, the physiological role of gliotransmission is highly debatable (see Nedergaard and Verkhratsky, 2012; Chai et al., 2017; Papouin et al., 2017; Fiacco and McCarthy, 2018; Savtchouk and Volterra, 2018), as gliotransmitter release has been reliably demonstrated only *in vitro* in cultures and brain slice experiments that are often accompanied by manipulations (e.g., high frequency stimulation) which can affect astrocytic channels or receptors leading to impaired signaling cascades. This experimental design imposes questions about the existence of gliotransmission (Wolosker et al., 2016; Chai et al., 2017) and whether it plays a physiological role in the brain (Fiacco and McCarthy, 2018). Although previous studies found no correlation between astrocytic Ca^2+^ signaling and gliotransmitter release (Fiacco et al., 2007; Petravicz et al., 2008; Agulhon et al., 2010), there is increasing evidence supporting the importance of both the GJ-mediated connectivity and function of astrocytic networks for neuronal-astrocytic communication and control of neuronal network activity (Covelo and Araque, 2016, 2018). Consequently, astrocytic alterations likely lead to aberrant modulation of both synaptic transmission and synchronization of network oscillations, which is also accompanied by changes in behavioral performance.

## Author Contributions

All authors conceived the project, wrote and approved the manuscript.

## Conflict of Interest

The authors declare that the research was conducted in the absence of any commercial or financial relationships that could be construed as a potential conflict of interest.
